# Digital health technologies in Primary Health Care in rural territories: A protocol for scoping review

**DOI:** 10.1371/journal.pone.0331902

**Published:** 2025-09-08

**Authors:** Vinicius Costa Maia Monteiro, Ísis de Siqueira Silva, Pedro Bezerra Xavier, Felipe Magdiel Bandeira Montenegro, Josemario de Abreu Silva, Severina Alice da Costa Uchoa

**Affiliations:** 1 Department of Collective Health, Federal University of Rio Grande do Norte, Natal, Rio Grande do Norte, Brazil; 2 Undergraduate Nursing Course, Santo André Educational Complex College, Açu, Rio Grande do Norte, Brazil; 3 Postgraduate Program in Family Health, Oswaldo Cruz Foundation, Natal, Rio Grande do Norte, Brazil; Federal University of São João del Rei, BRAZIL

## Abstract

**Aim:**

To describe the protocol for a scoping review on digital health technologies in Primary Health Care in rural territories, with a view to evaluating their impact on the attributes of Primary Health Care and identifying barriers and facilitators for its implementation.

**Background:**

Rural populations face significant barriers in accessing health care, and digital health emerges as a promising strategy to overcome challenges. Nonetheless, there is a gap in the literature regarding the systematic evaluation of the impact of these technologies on rural Primary Health Care, which justifies this scoping review.

**Method:**

Scoping review protocol, conducted according to the guidelines of the JBI Manual for Evidence Synthesis. It was duly registered in the Open Science Framework platform, with information on the conduct of the study in nine stages, following the PRISMA-ScR. The search strategy includes relevant databases and gray literature to ensure a broad mapping of scientific production on the topic. The analysis of quantitative variables will be carried out by simple descriptive statistics, with absolute and relative frequencies, while qualitative data will undergo thematic content analysis, following the stages of preparation, organization and reporting.

**Expected results:**

By evaluating the impact of digital technologies on rural Primary Health Care services, as well as identifying barriers and facilitators in their implementation, information is sought to improve access and quality of these services in rural territories through digital health.

**Conclusion:**

This review will have great practical relevance for managers, public policy makers, health professionals and researchers, as it is intended to map scientific evidence that can support decision-making and the development of strategies for the implementation of digital health in the studied context.

## Introduction

The World Health Organization (WHO) created the Global Initiative on Digital Health (GIDH) with the aim of supporting planning, offering resources and developing strong foundations for digital health transformation and equitably strengthening health systems. According to the WHO, digital health is defined as the use of information and communication technologies (ICTs) to support health services, surveillance, education, research and dissemination of health knowledge [1]. Digital health encompasses a broad spectrum of technological innovations, such as telemedicine, tracking devices, electronic health records, and emerging technologies, such as artificial intelligence and big data. The introduction and popularization of these technologies have transformed the provision and management of health services globally, with opportunities to expand access, optimize management, empower users and promote the well-being of populations [2].

In Primary Health Care (PHC), the incorporation of ICTs has shown potential to improve care, especially in developing countries, through resources such as telemedicine, electronic medical records and remote monitoring [3,4]. Studies highlight the relevance of these technologies to overcome structural and geographical limitations in low- and middle-income countries, such as those located in Africa, Latin America and Asia, where these resources have mitigated delays in diagnoses and public health interventions [5,6].

Rural areas, even in developed countries like the United States and New Zealand, face a number of difficulties in accessing health policies, such as the scarcity of medical services, geographical distances and difficulties in traveling to health centers due to the lack of adequate transportation [7,8]. Moreover, the precarious infrastructure and the limitation of qualified human resources aggravate the situation, which makes care less frequent and effective [9]. Inadequate communication about available services and misinformation also contribute to the under-utilization of resources. These structural barriers, added to socioeconomic factors, perpetuate inequalities and compromise the health of the rural population [10]. In the face of these challenges, digital health emerges as a potential tool to address the difficulties of PHC in rural and resource-limited areas. Available technologies enable patients in isolated communities to receive more agile and efficient care, reducing the need for long and costly commutes. In addition, they facilitate the continuous monitoring of chronic conditions, promote health education and strengthen communication between professionals and patients [11]. Accordingly, digital health can help to improve the quality of PHC services and increase their coverage in rural regions and, consequently, can reduce disparities in access to health care and promote greater equity in the health system [12].

In rural contexts, digital health solutions need to be specifically tailored to address the aforementioned challenges. First, telecommunications infrastructure is often limited, with low connectivity and unstable network coverage, which requires the development of platforms that operate properly in conditions of reduced internet. Moreover, familiarity with digital technologies may be lower among local residents and professionals, which imposes the need for intuitive interfaces and targeted training programs [6,7]. Technologies like mobile applications optimized for offline use, asynchronous telemedicine systems, and energy-efficient monitoring devices demonstrate potential to improve the quality of health care in rural areas [12].

The quality of health care is evaluated based on a set of fundamental characteristics: efficacy, effectiveness, efficiency, optimization, acceptability, legitimacy and equity [13]. In PHC, this quality is manifested through specific attributes, such as first contact, longitudinality, comprehensiveness, coordination, family approach and community focus [14]. With the incorporation of digital health technologies, these attributes require a customized evaluation model. Electronic record systems allow for more accurate longitudinal follow-up, while integrated platforms facilitate comprehensiveness and coordination by connecting different levels of care. Likewise, telemedicine and other digital solutions expand the first contact and the family approach, with the promotion of greater accessibility and better communication [15–17]. Thus, the evaluation of the quality of care intermediated by digital health in rural PHC requires a personalized model that includes both the predefined indicators and the structure, which incorporates resource elements; the process, which incorporates elements of activity; the results, which incorporates products, intermediate results; and the impacts, regarding the new dimensions introduced by technological innovations [15].

Despite technological advances, there is still a gap in the literature regarding the systematic evaluation of the impact of digital health technologies on PHC in rural areas, which justifies the performance of this scoping review [16]. One of the main limitations is the lack of research that evaluates the long-term effects of the implementation of these technologies, especially with regard to sustainability, user adoption and improvements in public health. In addition, research is mostly concentrated in urban or peri-urban regions, which leaves open how factors specific to rural areas, such as cultural diversity, socioeconomic conditions and geographic challenges, influence the success of these solutions [17].

There are few studies that address the particularities of rural populations, such as the elderly citizens and people without technological familiarity, and the impact of educational and digital barriers on the adoption of these tools [18]. In addition, the ethical and privacy aspects of the use of health data in areas where the technological infrastructure is precarious require further investigation [19,20]. A preliminary search conducted in the Cochrane Library (eHealth in All Text AND “Rural Population” in All Text OR “Rural Health Services” in All Text) identified 27 reviews on digital health technologies in PHC. Of these, only two specifically addressed the theme in rural populations [5,21]. In the Open Science Framework (OSF), no registered review protocols were identified on this specific topic. This scenario highlights the originality and relevance of a review that specifically explores the use of these technologies in rural territories.

### Aim

To describe the protocol for a scoping review on digital health technologies in Primary Health Care in rural territories, with a view to evaluating their impact on the attributes of Primary Health Care and identifying barriers and facilitators for its implementation.

## Method

### Study

This scoping review protocol was developed prior to the start of the data collection phase. The study is currently in the planning phase, with data collection expected to begin in July 2025 and be completed by August 2025. Data analysis and dissemination of results are expected to occur by October 2025. To mitigate the risk of data obsolescence due to the rapid pace of digital health innovations, we will consider continuously updating the database search throughout the study development period. This method is suitable for mapping the extent, scope and nature of a field of study, especially in emerging or complex areas, by identifying gaps in knowledge and synthesizing practices and challenges related to the topic [22]. The preparation of a detailed project is important to ensure the transparency of the process and minimize potential reporting biases, in order to contribute to the validity and reliability of the results [23]. The present research was developed according to the guidelines of the JBI Manual for Evidence Synthesis. The review will be conducted according to the framework originally proposed by Arksey and O’Malley [24], extended by Levac *et al*. (2010) [25], as presented in the updated version by Peters *et al*. (2020) in the manual released in 2024. [23] In addition, it will follow the Preferred Reporting Items for Systematic Reviews and Meta-Analyses – Scoping Review (PRISMA-ScR) [26].

**Fig 1:**
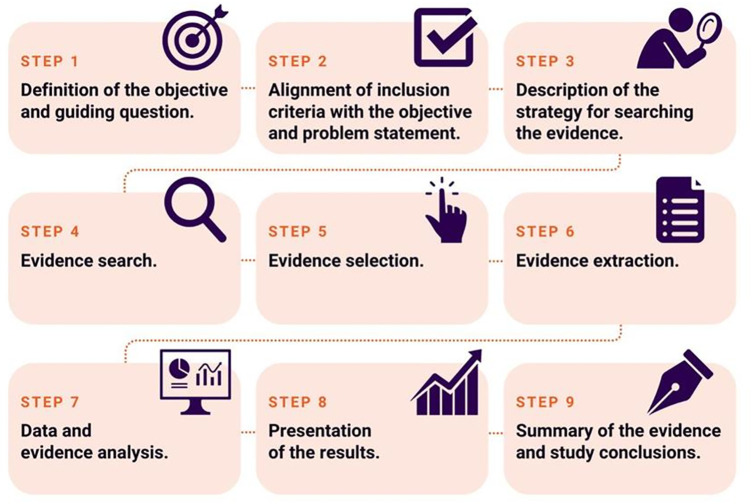
Stages to be developed in the scoping review.

**Registration** This protocol containing the planning for conducting the study was prepared and duly registered on the Open Science Framework platform, available at the following electronic address: https://osf.io/kzfx3/. [27] This registration provides information on the conduct of the study in nine stages (Fig 1), which will be detailed in the following sections.

#### Stage 1: Defining the research aims and questions.

The aims and guiding questions were determined based on the mnemonic PCC (Population, Concept and Context), where “P” will be represented by the population of rural territories; “C”, referring to the concept, encompasses digital health; and “C”, referring to the context, encompasses Primary Health Care. [23,26] Thus, it aims to: (1) identify the digital health interventions applied in this context, (2) characterize the geographical scenarios in which they are implemented, and (3) collect information on the impact of digital health on the quality of PHC in rural territories.

Based on this, the following guiding questions were listed: What digital health technologies are used in PHC in rural territories? In which geographic scenarios (countries) are these digital health technologies implemented in PHC in rural territories? What are the impacts of digital health on the quality of PHC in rural territories?

The descriptors and respective synonyms defined for this research are described in Table 1.

**Table 1:** Descriptors and their synonyms defined for the PCC mnemonic.

PCC mnemonic	Definition	Descriptors and synonyms (Medical Subject Headings – MESH)
**Population/Problem:** Population of rural territories	Segment of the population that lives in locations far from urban centers, characterized by a lower population concentration. [28]	Rural area; rural areas; rural space; rural environment; rural areas; Rural Settlement; Rural Settlements; Average Community; Small Community; Rural Community; Average Communities; Small Communities; Rural Communities; Rural Household; Rural Housing; Small Communities; Agricultural Population; Riverside Population; Agricultural Populations; Rural Populations; Rural Residence; Rural Residences
**Concept:** Digital health	Use of information and communication technology to support health services, surveillance, education, research and dissemination of knowledge in health. [1]	eHealth; e-Health; telehealth; Telecare; mHealth; Telerehabilitation; Telehomecare; home telehealth; Home telecare; telemonitoring; telecare monitoring system; telenursing; Digital Health; Digital Health Strategies; Digital Health Strategy; Digital Health Interventions; eHealth Strategies and Policies; Telemedicine; Rural Telemedicine; Telemedicine for Rural Areas; Telemedicine for the Rural Population; Telemedicine in Rural Areas; Telemedicine in Rural Hospitals; Telemedicine in Rural Settlements; Telemedicine in the Rural Environment; Telemedicine, Rural
**Context:** Primary Health Care	First level of health care, with an emphasis on people’s needs as soon as possible along the continuum, from health promotion and disease prevention, to treatment, rehabilitation and palliative care. [29]	Health, Rural; Rural Health Services; Center, Rural Health; Centers, Rural Health; Health Center, Rural; Health Centers, Rural; Health Service, Rural; Health Services, Rural; Rural Health Center; Rural Health Centers; Rural Health Service; Service, Rural Health; Services, Rural Health; health of the rural population

#### Stage 2: Aligning the inclusion criteria with the research aims and questions.

In order to select the studies from which information on digital health in rural areas will be extracted, the following eligibility criteria were established, as displayed in Table 2. There will be no delimitation of year of publication or language in searches in the different databases.

**Table 2:** Eligibility criteria for selection of studies.

Eligibility criteria	White/gray literature
**Inclusion criteria**	For white literature: Studies of quantitative, qualitative and/or mixed nature, with experimental, observational design and/or reviews For gray literature: Government manuals and technical notes, theses and dissertations
**Exclusion criteria**	For white literature: Studies focused exclusively on urban and/or peri-urban contexts, studies that address digital health technologies outside the context of PHC, studies that address only technical aspects of technologies without evaluating the impact on PHC quality, opinion articles, letters to the editor and editorials without empirical support For gray literature: Studies without methodological foundation, experience reports, summaries in annals of events, institutional reports without explicit methodology, documents without proof of scientific rigor, books and other physical materials

#### Stage 3: Describing the planned approach to searching for evidence.

In order to identify the descriptors, the Medical Subject Headings (MeSH) vocabularies were used. According to the mnemonic strategy, the terms “Rural Health”, “Rural Health Services” and “Rural Population” were used for the population; the descriptor “Digital Health” and other synonyms were used for the concept; and the search filter (University Flinders, [202-]) was adapted for the PHC context, as displayed in Table 3.

**Table 3:** Description of the strategy for searching for evidence*.

#	Searches
**1**	“Rural health”[mh] OR “Rural health services”[mh] OR “Rural population”[mh]
**2**	Line for the issue of Rural community #1
**3**	“Primary health care”[tiab] OR “general practi*”[tiab] OR “primary care”[tiab] OR “Family practice”[mh] OR “Community health services”[mh] OR “Community health nursing”[mh] OR “Community health workers”[mh] OR “Community mental health services”[mh] OR “Community pharmacy services”[mh] OR “Home care services”[mh] OR “preventive health services”[mh] OR “preventive service*”[tiab] OR “Preventive health”[tiab] OR “Health promotion”[tiab] OR “Family health program”[tiab] OR “Family health strategy”[tiab]
**4**	Line for the issue of Primary Health Care #3
**5**	telemedicine[mh] OR telemed*[tiab] OR Ehealth[tiab] OR “e-Health”[tiab] OR Telerehabilitation[mh] OR Telereh*[tiab] OR telehealth[tiab] OR Telecare[tiab] OR mHealth[tiab] OR “home telehealth”[tiab] OR “Home telecare”[tiab] OR “virtual rehabilitation*”[tiab] OR telemonitoring*[tiab] OR “telecare monitoring system”[tiab] OR telenursing[tiab] OR “Digital Health”[tiab] OR “digital health strateg*”[tiab] OR “Digital Health Interventions”[tiab] OR “Virtual Medicine”[tiab] OR “information and communication Technolog*”[tiab]
**6**	Line for the issue of Digital health #5
**7**	#1 and #3 and #5 Line combining the PCC mnemonic (result = 949)

*Research conducted on 11/01/2024 via MEDLINE/Pubmed.

#### Stage 4: Searching for evidence.

The search strategy used for each database is displayed in Table 4. The English language was used to structure the research strategy, since it is the main language in the scientific field [30]. Initially, the search for evidence will be carried out in the following portals and databases: MEDLINE/PubMed, Embase, Web of Science, Scopus and LILACS. A second phase of search will be conducted in Google Scholar, Open Grey, ProQuest Dissertations and Theses Global and Preprints for Health Sciences [medRXiv] and CAPES Thesis and Dissertation Bank to identify gray literature. In addition, the publications present in the reference lists of the documents included in the search phases mentioned may be added to the sample. If necessary, the corresponding authors will be contacted for additional information support.

**Table 4:** Search strategy for data sources.

Data source	Search strategy
MEDLINE/PubMed	(“Rural health”[MeSH Terms] OR “Rural health services”[MeSH Terms] OR “Rural population”[MeSH Terms]) AND (“Primary health care”[Title/Abstract] OR “general practi*”[Title/Abstract] OR “primary care”[Title/Abstract] OR “Family practice”[MeSH Terms] OR “Community health services”[MeSH Terms] OR “Community health nursing”[MeSH Terms] OR “Community health workers”[MeSH Terms] OR “Community mental health services”[MeSH Terms] OR “Community pharmacy services”[MeSH Terms] OR “Home care services”[MeSH Terms] OR “preventive health services”[MeSH Terms] OR “preventive service*”[Title/Abstract] OR “Preventive health”[Title/Abstract] OR “Health promotion”[Title/Abstract] OR “Family health program”[Title/Abstract] OR “Family health strategy”[Title/Abstract]) AND (“telemedicine”[MeSH Terms] OR “telemed*”[Title/Abstract] OR “Ehealth”[Title/Abstract] OR “e-Health”[Title/Abstract] OR “telerehabilitation”[MeSH Terms] OR “telereh*”[Title/Abstract] OR “telehealth”[Title/Abstract] OR “Telecare”[Title/Abstract] OR “mHealth”[Title/Abstract] OR “home telehealth”[Title/Abstract] OR “Home telecare”[Title/Abstract] OR “virtual rehabilitation*”[Title/Abstract] OR “telemonitoring*”[Title/Abstract] OR “telecare monitoring system”[Title/Abstract] OR “telenursing”[Title/Abstract] OR “Digital Health”[Title/Abstract] OR “digital health strateg*”[Title/Abstract] OR “Digital Health Interventions”[Title/Abstract] OR “Virtual Medicine”[Title/Abstract] OR “information and communication Technolog*”[Title/Abstract])
Embase	(‘rural health’/exp OR ‘rural health care’/exp OR ‘rural population’/exp) AND (‘primary health care’:ti,ab OR ‘general practice’:ti,ab OR ‘general practices’:ti,ab OR ‘primary care’:ti,ab OR ‘general practice’/exp OR ‘community care’/exp OR ‘community health nursing’/exp OR ‘health auxiliary’/exp OR ‘community mental health service’/exp OR ‘pharmacy (shop)’/exp OR ‘home care’/exp OR ‘preventive health service’/exp OR ‘preventive service’:ti,ab OR ‘preventive services’:ti,ab OR ‘preventive health’:ti,ab OR ‘health promotion’:ti,ab OR ‘family health program’:ti,ab OR ‘family health strategy’:ti,ab) AND (‘ehealth’:ti,ab OR ‘e-health’:ti,ab OR ‘telehealth’:ti,ab OR ‘telecare’:ti,ab OR ‘mhealth’:ti,ab OR ‘home telehealth’:ti,ab OR ‘home telecare’:ti,ab OR ‘virtual rehabilitation’:ti,ab OR telemonitoring*:ti,ab OR ‘telecare monitoring system’:ti,ab OR ‘telenursing’:ti,ab OR ‘digital health’:ti,ab OR ‘digital health strategy’:ti,ab OR ‘digital health strategies’:ti,ab OR ‘digital health interventions’:ti,ab OR ‘telemedicine’/exp OR telemed*:ti,ab OR ‘virtual medicine’:ti,ab) AND [embase]/lim
*Web of Science*	(“Primary health care” OR “general practi*” OR “primary care” OR “Family practice” OR “Community health services” OR “Community health nursing” OR “Community health workers” OR “Community mental health services” OR “Community pharmacy services” OR “Home care services” OR “preventive health services” OR “preventive service*” OR “Preventive health” OR “Health promotion” OR “Family health program” OR “Family health strategy”) AND (Ehealth OR “e-Health” OR “telehealth” OR “Telecare” OR “mHealth” OR “home telehealth” OR “Home telecare” OR “virtual rehabilitation*” OR telemonitoring* OR “telecare monitoring system” OR “telenursing” OR “Digital Health” OR “digital health strateg*” OR “Digital Health Interventions” OR telemed* OR “Virtual Medicine” OR “information and communication Technolog*”) (Topic) AND (“Rural health” OR “Rural health services” OR “Rural population” OR Rural) (Title)
Scopus	TITLE (“Rural health” OR “Rural health services” OR “Rural population” OR rural)) AND TITLE-ABS-KEY (“Primary health care” OR “general practi*” OR “primary care” OR “Family practice” OR “Community health services” OR “Community health nursing” OR “Community health workers” OR “Community mental health services” OR “Community pharmacy services” OR “Home care services” OR “preventive health services” OR “preventive service*” OR “Preventive health” OR “Health promotion” OR “Family health program” OR “Family health strategy”) AND (ehealth OR “e-Health” OR “telehealth” OR “Telecare” OR “mHealth” OR “home telehealth” OR “Home telecare” OR “virtual rehabilitation*” OR telemonitoring* OR “telecare monitoring system” OR “telenursing” OR “Digital Health” OR “digital health strateg*” OR “Digital Health Interventions” OR telemed* OR “Virtual Medicine” OR “information and communication Technolog*”)
LILACS	(mh:“População Rural” OR “Assentamento Rural” OR “Comunidade Rural” OR “Comunidade Rurais” OR “Rural health” OR “População Ribeirinha” OR mh:”Saúde da População Rural” OR “Rural health services” OR “Rural population” OR mh:”Serviços de Saúde Rural” OR “Serviços Rurais de Saúde” OR rural) AND (mh:”Atenção Primária à Saúde” OR “Primary health care” OR “general practice” OR “primary care” OR “Atendimento Primário de Saúde” OR “Cuidado de Saúde Primário” OR “Cuidado Primário de Saúde” OR “Atenção Básica” OR “Atenção Básica à Saúde” OR mh:”Medicina de Família e Comunidade” OR “Family practice” OR “Medicina Familiar” OR mh:”Serviços de Saúde Comunitária” OR “Assistência à Saúde Comunitária” OR “Community health services” OR mh:”Enfermagem em Saúde Comunitária” OR “Enfermagem Comunitária” OR “Community health nursing” OR mh:”Agentes Comunitários de Saúde” OR ACS OR “Community health workers” OR mh:”Serviços Comunitários de Saúde Mental” OR “Community mental health services” OR mh:”Serviços Comunitários de Farmácia” OR “Community pharmacy services” OR “Home care services” OR mh:”Serviços Comunitários de Farmácia” OR “preventive health services” OR “preventive service” OR “Programas Preventivos” OR “Preventive health” OR “Health promotion” OR “Family health program” OR “Family health strategy” “Estratégia Saúde da Família” OR mh:”Estratégias de Saúde Nacionais” OR “Programa Saúde da Família” OR “estratégia de saúde da família”) AND (mh:”Telemedicina para as Zonas Rurais e Remotas” OR “Telemedicina em Assentamentos Rurais” OR “Telemedicina em Zonas Rurais” OR mh:Telemedicina OR telemedicine OR telemed* OR Ehealth OR “e-Health” OR mh:Telerreabilitação OR telerehabilitation OR telereh* OR telehealth OR Telecare OR mHealth OR “home telehealth” OR “Home telecare” OR “virtual rehabilitation” OR “virtual rehabilitations” OR telemonitoring* OR “telecare monitoring system” OR telenursing OR “Digital Health” OR mh:”Saúde Digital” OR “digital health strategy” “Tecnologia de Saúde Digital” OR “Digital Health Interventions” OR mh:”Estratégias de eSaúde” OR “Estratégia de eSaúde” OR “Virtual Medicine” OR Telessaúde OR “information and communication Technology” OR “tecnologias da informação e da comunicação” OR remot*)

A pilot test of the search strategy will be carried out in MEDLINE/PubMed for white literature and in Google Scholar for gray literature, in order to verify possible limitations in the search and identification of studies.

#### Stage 5: Selecting the evidence.

The selection of studies will be guided by the steps proposed in the PRISMA-ScR for white and gray literature, namely: (1) identification; (2) screening; (3) eligibility; and (4) inclusion, and will be presented in the review selection diagram. The process of selecting gray literature will follow the recommended guidelines, with specific strategies for searches in Google Scholar repositories and other sources [31]. After identifying the studies in the data sources, the studies will be entered into the Endnote management software and all duplicates will be removed. The screening of studies will be carried out by evaluation by titles and abstracts based on the aforementioned inclusion criteria, using the Rayyan® software, which will help in blinding the data between the two reviewers involved in it. Any disagreements between the two reviewers will be discussed with a third party. The selected studies will be retrieved, read in full, and exported to a database maintained in the Microsoft® Excel® for Microsoft 365 MSO program (Version 2412 Build 16.0.18324.20092). With the definition of the final sample, the reasons for exclusion will be highlighted, when necessary, to ensure transparency in the evidence selection process.

#### Stage 6: Extracting the evidence.

For the extraction process, a data collection form adapted according to Peters *et al*. will be used (2020), S1 Table [23]. The form consists of two parts: characterization of the study (title, authors, journal, year, country, language, study design/type of literature and main results) and data related to the research questions (type of digital health technology, geographic scenarios – countries and contexts, and impacts of digital health on the quality of rural PHC). Regarding the assessment of the quality of care mediated by digital health in rural PHC, the criteria mentioned in the introduction of our study, proposed by the model by Silva et al. (2024), will be considered [15]. Previously, a pilot test will be carried out, in order to refine the form and ensure extraction of all relevant data. It will be evaluated in a random sample of 25 articles, with analysis of titles and abstracts in a data source. Subsequently, necessary adjustments will be made to the form [23].

#### Stage 7: Analyzing the evidence.

The analysis of the evidence will be carried out according to the following steps: (1) data analysis; (2) presentation of the results to address the guiding questions; and (3) interpretation of the implications of the results for other studies and services. The analysis of quantitative variables – such as journals, year of publication, country, language, study design and type of digital health technology – will be conducted by simple descriptive statistics, presented in absolute and relative frequencies to allow the identification of recurrent variables. In order to perform this analysis, the statistical software SPSS, version 20.0, will be used [32]. Regarding qualitative data – such as the main results of the studies, countries and contexts and the impacts of digital health on the quality of Primary Health Care – thematic content analysis will be carried out, following the following steps: I) preparation, with the initial immersion of the data focused on surveying the implications of the studies, geographical and contextual aspects and impacts on the quality of care; II) organization, with the identification of preliminary codes and the definition of thematic categories; and III) report, with the interpretation of the findings based on the implications of the studies [33].

The synthesis of the descriptive results will be presented through tables, thematic maps and diagrams for better visualization of the results along with the narrative summary of the mapped data. Among the graphic elements that will be presented, a map of countries that use digital health technologies in PHC in rural communities will be elaborated, based on the analyzed publications, using the GeoDa software, version 1.20. (*Center for Spatial Data Science*, Chicago, IL, EUA).

#### Stage 8: Presenting the results and consulting with experts.

A preliminary final report will be produced based on the PRISMA-ScR guidelines [26], including the data collected in the previous stage in the formats of flowcharts, figures and/or graphs. These preliminary results will be presented to a panel of stakeholders with expertise in PHC and digital health. Consultation with these stakeholders is interesting for planning health innovations, especially for policies, services and delivery methods [34]. In addition, this stage will facilitate the sharing of results and the suggestion of means for their dissemination.

Accordingly, 12 stakeholders will be determined by the snowball method, namely: researchers (3), health professionals (3) and digital professionals (3) – all with experience in digital health focused on PHC, in addition to (3) PHC managers. The first to be included will be identified by the researchers of this study, who will successively indicate the next ones. The invitation to participate will be made through e-mail, with information about the objectives of the study, the research questions and the preliminary results. Stakeholders will not be identified, and the authors will request the appreciation of the presented results, with the aim of improving the obtained evidence, strengthening the rigor of the findings and incorporating qualified perspectives into the analyzed content, as well as possible new fields of study and recommendations for future research.

#### Stage 9: Summarizing the evidence regarding the purpose of the review and drawing conclusions.

The main results will be summarized with an overview of the topic, concepts and specifications of the available evidence, in addition to resuming the aims and questions of the study research. The implications found on the use of digital health technologies in PHC in rural populations will be presented and discussed in order to collaborate with discussions on the topic.

### Ethical aspects

Approval from the research ethics committee is not required because the study will use data already available in the public domain. The consultation processes to be carried out use the scientific knowledge of experts, that is, it is a technical and voluntary activity. Although ethical consideration is not necessary, the study will respect the ethical guidelines for research, with transparency and sharing of findings in open access.

## Results

By evaluating the impact of digital technologies on rural PHC services, specifically in relation to improving access, continuity, coordination and comprehensiveness of care, as well as identifying barriers and facilitators in their implementation, this review will seek information to improve access and quality of these services in rural territories through digital health.

It is expected that the results of this review will contribute substantially to the advancement of knowledge on the subject under study and provide scientific support for future health interventions and public policies. The identification of digital health technologies and their applications in the rural context can increase understanding of the impacts of these innovations, as well as assist in the strategic planning of actions that expand access and improve the quality of services provided in PHC. In addition, it is hoped that the presentation of the results to qualified stakeholders will promote important discussions with further contributions to the study.

## Discussion

The implementation of digital health technologies in PHC in rural areas presents an emerging and promising field, especially in contexts characterized by challenges in access to health care, such as limited infrastructure, scarcity of professionals and geographical distances. This protocol highlights that the extent of the applicability of digital health technologies in rural territories remains underexplored [35].

Among the limitations identified, the dependence on data available in the literature stands out, which may not fully reflect the reality in regions of difficult access. However, based on scientific literature, it will be possible to identify gaps and contexts that require further field research. From the preliminary search carried out, limitations in obtaining specific literature on rural areas are evident, as well as the lack of uniformity in the definition of “rural area” in the identified studies. To minimize these difficulties, a search with a comprehensive design of the eligibility criteria for studies, the use of descriptors and their respective synonyms in the search strategy and the choice of relevant data sources. To address the limitation regarding the definition of “rural area,” the definitions presented in Table 1 will be considered.

In addition, the scoping review does not evaluate the quality of the included studies, which limits detailed conclusions about the analyzed interventions. To deepen these issues, it is suggested to carry out systematic reviews and meta-analyses, as well as experimental and observational studies that explore the practical implementation of digital technologies in rural territories.

## Conclusion

The expected results of this review have great practical relevance, as it is intended to map scientific evidence that can support decision-making and the development of strategies for the implementation of digital health in rural PHC. By identifying viable technologies, barriers and facilitators to their adoption, the review will allow for more assertive planning, guided by data from the literature, to expand access and improve the quality of health services in these regions. For managers, the findings can help in the allocation of resources, in the definition of priorities for investments in innovation in health, as well as in the formulation of guidelines that optimize the integration of technologies in rural PHC services. For public policy makers, the results can support the creation of programs and incentives aimed at expanding digital health in PHC in rural territories.

For rural PHC professionals, the results can provide subsidies for care practice mediated by digital health in similar regions and contexts. For researchers, the results can help in the directions for future studies.

## Supporting information

S1 Table Data extraction form adapted from Peters *et al*. (2020). (DOCX)

S2 Digital Health_PRISMA-P-checklist. (PDF)
