# Supplementary material for: Digital health technologies in Primary Health Care in rural territories: A protocol for scoping review
Source: PLoS One. 2025 Sep 8;20(9):e0331902. doi: 10.1371/journal.pone.0331902 (PMC12416644; doi:10.1371/journal.pone.0331902)
Supplement: S1 Table — (DOCX) [file pone.0331902.s001.docx]

**S1 Table. Data extraction form adapted from Peters et al. (2020)**

| **Study characteristics** | |
| --- | --- |
| Study title |  |
| Authors |  |
| Journal |  |
| Year of publication |  |
| Study country |  |
| Language |  |
| Study design/type of file |  |
| Study objective |  |
| Main results |  |
| **Research question data** | |
| Type of digital health technology |  |
| Geographic settings (countries and contexts) |  |
| Impacts of digital health on healthcare quality |  |
